# Nutritional Supplementation for Myopia Prevention and Control: A Systematic Review of Randomized Controlled Trials

**DOI:** 10.3390/nu18010004

**Published:** 2025-12-19

**Authors:** Clara Martinez-Perez, Ana Paula Oliveira

**Affiliations:** 1 Applied Physics Department (Optometry Area), Facultade de Óptica e Optometría, Universidade de Santiago de Compostela, 15705 Santiago de Compostela, Spain; 2 Instituto Superior de Educação e Ciências de Lisboa (ISEC Lisboa), Alameda das Linhas de Torres, 179, 1750-142 Lisboa, Portugal; ana.oliveira@iseclisboa.pt; 3 Centro de Investigação, Desenvolvimento e Inovação em Turismo (CiTUR)—Polo Estoril, Avenida Condes de Barcelona, n.° 808, 2769-510 Estoril, Portugal

**Keywords:** myopia, nutritional supplementation, carotenoids, anthocyanins, randomized controlled trials

## Abstract

Background/Objectives: Nutritional supplementation has been proposed as a potential adjunct strategy in myopia prevention and control through antioxidative, anti-inflammatory, and extracellular matrix-regulating mechanisms. This systematic review aimed to evaluate randomized controlled trial (RCT) evidence on the effects of carotenoids, anthocyanins, polyunsaturated fatty acids, and combined nutraceutical formulations on refractive outcomes, axial length, macular pigment optical density (MPOD), visual function, and symptoms of visual fatigue. Methods: The review was registered in PROSPERO (CRD420251149727) and conducted in accordance with PRISMA 2020 and AMSTAR-2 guidelines. PubMed, Web of Science, and Scopus were searched up to 5 August 2025. Eligible studies were RCTs involving individuals with myopia or at risk of myopia, comparing nutritional supplementation with placebo or active controls. Two reviewers independently screened studies, extracted data, and assessed risk of bias using the Cochrane RoB 2 tool. Results: Nine RCTs were included. Carotenoids such as crocetin, lutein, zeaxanthin, and astaxanthin produced modest benefits, including improved MPOD, reduced visual fatigue, and—in one pediatric trial—slightly less axial elongation. Anthocyanin-rich extracts improved mesopic contrast sensitivity and subjective asthenopia. A combined carotenoid–polyphenol formulation enhanced accommodative facility. However, no consistent clinically meaningful reduction in myopia progression was observed. Trials were generally small, heterogeneous, and short in duration. Conclusions: Nutritional supplementation may improve visual function and retinal antioxidant status but lacks strong evidence for slowing myopia progression. Larger, long-term RCTs are needed before recommending supplementation for routine myopia management.

## 1. Introduction

Myopia has emerged as a leading cause of visual impairment worldwide, with projections suggesting that nearly half of the global population will be affected by 2050 [1]. Beyond refractive error, high myopia predisposes individuals to severe complications such as retinal detachment, glaucoma, and myopic maculopathy [2], underscoring the urgent need for effective preventive and therapeutic strategies. While pharmacological (e.g., atropine) and optical interventions (e.g., orthokeratology, defocus lenses) remain the mainstay of myopia control [3], growing attention has turned to the potential role of nutritional supplementation in modulating disease onset and progression. Nutritional agents may exert beneficial effects by attenuating oxidative stress, regulating inflammatory pathways, and influencing scleral remodeling—processes all implicated in the pathogenesis of myopia [4,5].

Carotenoids and other nutritional factors have been proposed as potential modifiers of myopia development through antioxidative, anti-inflammatory, vascular, and extracellular matrix–related pathways. Macular xanthophylls such as lutein and zeaxanthin accumulate in the retina and contribute to retinal antioxidant defense and visual function [6,7,8,9]. Other fat-soluble vitamins and lipids, including vitamins A, E, and D, as well as polyunsaturated fatty acids (PUFAs), have been implicated in ocular growth regulation and retinal or choroidal homeostasis, mainly based on experimental and observational evidence [10,11,12,13,14,15,16,17]. However, findings from human studies remain inconsistent across populations and regions, with reported associations varying according to study design, exposure assessment, lifestyle confounding, and outcome definitions [15,18,19,20,21].

Taken together, current evidence suggests that nutrition may influence ocular structure and function through antioxidative, anti-inflammatory, vascular, and ECM-regulatory pathways. However, much of this evidence originates from observational studies, animal experiments, or small pilot trials. To date, randomized controlled trials (RCTs) evaluating nutritional supplementation in myopia are limited in number, heterogeneous in design, and generally underpowered to guide clinical recommendations.

Therefore, the aim of this systematic review was to synthesize the available RCT evidence on nutritional supplementation for the prevention or control of myopia, with interventions conceptually grouped into carotenoids, anthocyanin-rich extracts, polyunsaturated fatty acids, and combined nutraceutical formulations. The review focuses on refractive outcomes, axial length, visual performance, and symptoms of asthenopia. By critically evaluating the current RCT evidence base, we also aim to identify knowledge gaps and outline priorities for future clinical research in this emerging field.

## 2. Materials and Methods

### 2.1. Research Question and PICOS Framework

**Figure 1:**
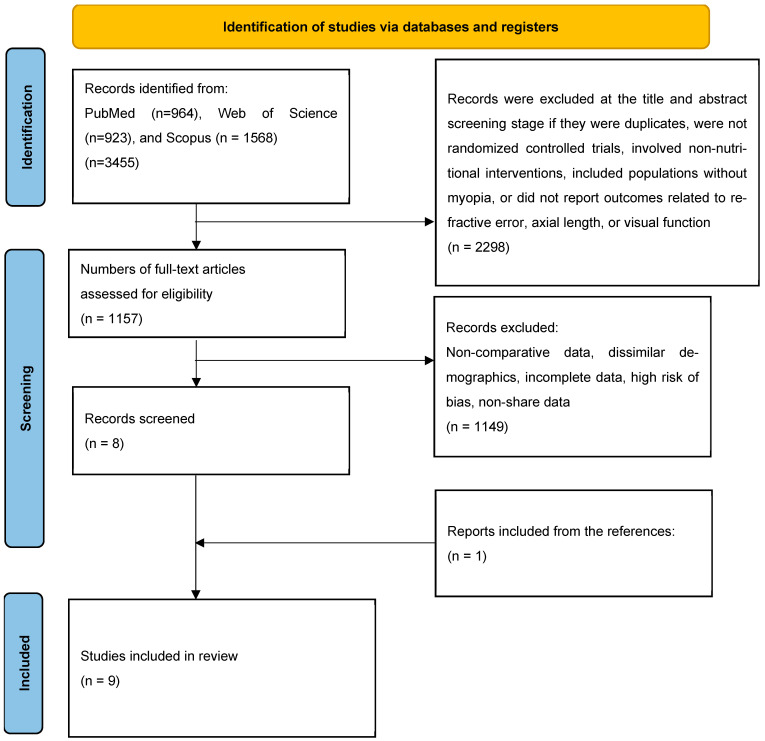
PRISMA flow diagram of study selection.

This systematic review was registered in PROSPERO (registration number: CRD420251149727) and conducted and reported in accordance with the PRISMA 2020 guidelines [22] and AMSTAR-2 methodological standards [23] (see Figure 1). A completed PRISMA 2020 checklist is available as Supplementary Material (Additional File S1). The final literature search was completed on 5 August 2025.

The research question was formulated using the PICOS framework to ensure methodological rigor and clinical relevance. A concise summary of the PICOS components is provided in Table 1. Briefly, we examined whether individuals with myopia or at risk of developing myopia experience improvements in visual performance, ocular structure, or symptoms of asthenopia when treated with nutritional supplementation, compared with placebo or alternative formulations, based on evidence from randomized controlled trials. Participants considered “at risk of developing myopia” were those defined as such within the original randomized controlled trials. These definitions varied across studies and typically included pre-myopic refractive status, age-based risk categories, family history of myopia, or functional and biometric indicators of increased risk, such as accommodative lag or baseline ocular growth parameters. No additional eligibility criteria were imposed beyond those specified in the original trial designs.

The primary outcomes were changes in refractive error, axial length, and macular pigment optical density, while secondary outcomes included visual acuity, contrast sensitivity, accommodative function, and patient-reported visual fatigue. Subgroup analyses by age, supplement category, and intervention duration were considered descriptively to explore heterogeneity.

### 2.2. Eligibility Criteria

Studies were excluded if they met any of the following criteria: case reports, case series, quasi-experimental or uncontrolled studies; systematic or narrative reviews; or duplicate publications from the same dataset. Additional exclusions were applied to studies rated as having a high risk of bias or insufficient methodological rigor, as well as those with incomplete or non-comparable demographic data. Trials were also excluded if they did not enroll participants with myopia or those at risk of developing myopia; if they lacked a randomized controlled design with a placebo or active comparator; or if they failed to report relevant outcomes, including refractive error, axial length, macular pigment optical density, visual function, or visual fatigue. Studies were also excluded when outcome data were insufficient to permit meaningful extraction or cross-trial comparison.

### 2.3. Information Sources

A comprehensive and systematic literature search was conducted across three major electronic databases: PubMed, Web of Science, and Scopus, with no restrictions on publication date or language. To maximize completeness, the reference lists of all included articles were also manually screened to identify additional relevant trials not captured in the initial database search.

### 2.4. Search Methods for Identification of Studies

The search strategy combined controlled vocabulary and free-text terms related to myopia and nutritional or dietary supplementation. Core search concepts included myopia-related outcomes (e.g., myopia, spherical equivalent, axial length) combined with nutrition- and supplementation-related terms. To ensure specificity, non-nutritional interventions (e.g., optical, pharmacological, or surgical myopia control strategies) were excluded using Boolean operators. Equivalent search strategies were applied to PubMed, Web of Science, and Scopus. The final search was completed on 11 September 2025. Full search strings for each database are provided in the Supplementary Material (Additional File S2).

The search strategy included nutrition-related terms covering a broad range of dietary supplements. Studies evaluating nutraceuticals, herbal preparations, or traditional medicine without randomized controlled designs or myopia-specific outcomes were excluded during screening. No publication type or study design filters were applied at the search stage; randomized controlled trials were identified during screening according to predefined PICOS criteria. The search strategy was developed and independently reviewed by two authors; a formal PRESS or PRISMA-S peer review was not conducted.

Two reviewers independently screened titles, abstracts, and full texts for eligibility, with disagreements resolved by consensus. Randomized controlled trials were identified during the screening and eligibility assessment stages in accordance with the predefined PICOS criteria.

### 2.5. Data Extraction and Data Items

Two authors (A.P.O. and C.M.P.) independently extracted data from all eligible randomized controlled trials. For each included study, the following key characteristics were recorded: first author’s name, year of publication, country or region, study design, sample size for each intervention and comparator group, mean age of participants, treatment modality (type of nutritional supplement), treatment duration, outcome measures (e.g., refractive error, axial length, macular pigment optical density, visual function, visual fatigue), follow-up schedule, and reported conflicts of interest. Any discrepancies in data extraction or eligibility assessment were resolved through discussion and consensus, without the need for a third reviewer. Study management, including duplicate removal and tracking of eligibility decisions, was performed using Rayyan (Rayyan Systems Inc., Qatar Computing Research Institute, Doha, Qatar).

The primary variables extracted focused on changes in refractive error, axial length, and macular pigment optical density across different follow-up periods. Additional data included visual function tests (visual acuity, contrast sensitivity, stereopsis, accommodative parameters), patient-reported outcomes (e.g., asthenopia questionnaires), supplement composition (active compounds, dosage, formulation, administration route), and study-level descriptors such as inclusion/exclusion criteria and subgroup characteristics (e.g., baseline age, refractive error severity, axial length). These data were collected to enable comparison of intervention protocols and to assess methodological heterogeneity across studies.

### 2.6. Methodological Quality and Risk of Bias Assessment

**Figure 2:**
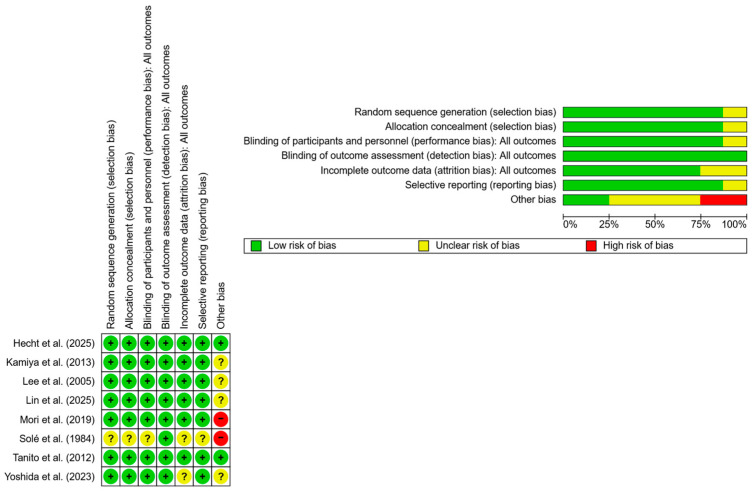
Risk of bias assessment (green = low risk; red = high risk; yellow = unknown) of 9 RCTs.

The methodological quality and risk of bias of the included randomized controlled trials were independently evaluated by two reviewers using the Cochrane Collaboration’s Risk of Bias tool (RoB 2). This tool evaluates potential bias across key domains: random sequence generation, allocation concealment, blinding of participants and personnel (performance bias), blinding of outcome assessment (detection bias), incomplete outcome data (attrition bias), selective reporting (reporting bias), and other potential sources of bias. Each domain was rated as low, high, or unclear risk according to predefined criteria. Discrepancies were resolved through discussion and consensus. The overall results of the risk of bias assessment are summarized in Figure 2, with detailed domain-specific justifications provided in Supplementary Material (Additional File S3).

### 2.7. Data Synthesis

In accordance with PRISMA 2020 recommendations, a quantitative meta-analysis was not performed due to the small number of eligible RCTs (n = 9) and substantial heterogeneity across interventions, dosages, populations, outcome measures, and follow-up periods. Outcomes were reported using non-comparable metrics, including axial length (mm), refractive error (diopters), macular pigment optical density, and symptom-based questionnaires, precluding meaningful statistical pooling.

Accordingly, a structured qualitative synthesis was conducted. Interventions were grouped into carotenoids, anthocyanin-rich extracts, polyunsaturated fatty acids, and combined nutraceutical formulations, and findings were summarized narratively, emphasizing the direction and consistency of effects. Descriptive visualizations were used solely to illustrate heterogeneity in study characteristics and outcome trends. Where available, standardized mean differences (SMDs) were calculated at the individual-study level for descriptive normalization across outcome scales and were not pooled or interpreted inferentially.

An exploratory funnel plot was generated (Additional File S4). Given the limited number of included trials and the absence of pooled effect estimates, publication bias was assessed qualitatively.

Given the absence of quantitative synthesis, no formal summary effect measures were estimated. Formal subgroup, heterogeneity, or sensitivity analyses were not conducted, as no quantitative synthesis was performed.

## 3. Results

### 3.1. Study Selection

Study selection followed the PRISMA 2020 flow diagram shown in Figure 1. A total of 3455 records were initially retrieved from PubMed (n = 964), Web of Science (n = 923), and Scopus (n = 1568) (Figure 1). After removal of duplicates and screening of titles and abstracts, 2298 records were excluded. Exclusion criteria at this stage included studies that were not randomized controlled trials, involved non-nutritional interventions, included populations without myopia, or did not report outcomes related to refractive error, axial length, or visual function. Subsequently, a total of 1157 full-text articles were assessed for eligibility. Of these, 1149 were excluded due to non-comparative data, non-comparable demographics, incomplete data, high risk of bias, or lack of shareable data. One additional study was identified through manual review of reference lists. In total, 9 studies met the inclusion criteria and were included in the systematic review [24,25,26,27,28,29,30,31,32].

### 3.2. Study Characteristics

Table 2 summarizes the main characteristics of the RCTs included in this review, which examined the effects of various nutritional supplements on visual performance, ocular structure, or symptoms of asthenopia across different populations and geographic settings, including Japan, China, Taiwan, South Korea, India, and France. Sample sizes varied considerably, from small single-center pilot studies with as few as 22 participants to multicenter trials encompassing nearly 100 eyes. Participant age also showed considerable variation, ranging from pediatric cohorts (6–12 years) to middle-aged adults (up to 53 years).

All studies employed a randomized controlled design and compared active supplementation with placebo or alternative formulations. The interventions included carotenoids (lutein, zeaxanthin, crocetin, astaxanthin, β-carotene), anthocyanin-rich extracts (bilberry, blackcurrant, grape), and, administered as capsules, tablets, or functional foods. Treatment durations ranged from very short-term protocols of 4 weeks to longer interventions lasting up to 6 months.

Outcome measures were heterogeneous, encompassing subjective questionnaires (Computer Vision Syndrome Questionnaire [CVS-Q], Visual Fatigue Likert Scale), functional tests (visual acuity, stereopsis, contrast sensitivity, amplitude of accommodation, accommodative facility), and structural parameters (axial length, refraction, macular pigment optical density, choroidal thickness). Several studies also applied objective physiological assessments, such as electroretinography, pupillary response tracking, or resonance Raman spectroscopy. Follow-up intervals varied from weekly measurements in shorter trials to multi-visit designs with assessments at 3 or 6 months.

Conflict of interest declarations were consistently reported across studies. Most trials indicated no financial or proprietary conflicts, although a minority acknowledged industry involvement, either through supplement provision or study funding.

**Figure 3:**
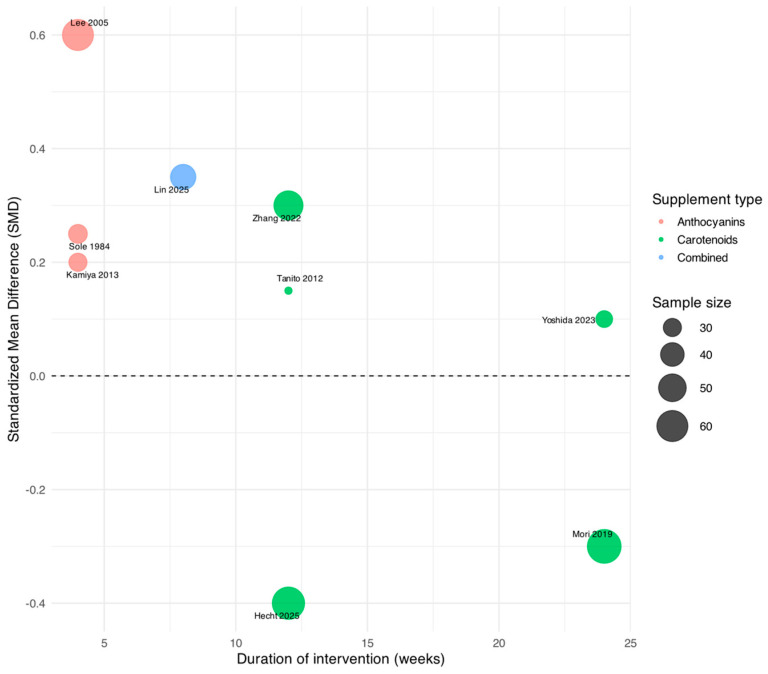
Bubble plot of nutritional supplementation trials in myopia.

To provide an overview of variability in study designs, Figure 3 presents a bubble plot illustrating trial duration against standardized mean differences (SMDs), with bubble size proportional to sample size and color coding according to supplement category. SMDs were calculated at the individual-study level to allow descriptive normalization across heterogeneous outcome measures. The figure highlights heterogeneity across trials, with shorter studies (4–8 weeks) primarily assessing functional outcomes and longer trials (12–24 weeks) focusing on structural parameters such as axial length and macular pigment optical density.

### 3.3. Risk of Bias Assessment

The risk of bias of the nine included randomized controlled trials was assessed using the Cochrane RoB 2 tool (Figure 2). Overall, most studies were judged to have a low risk of bias or some concerns across the evaluated domains. Random sequence generation and allocation concealment were generally adequate, with the majority of trials reporting appropriate randomization procedures. Blinding of participants and personnel was adequately described in several studies; however, some trials presented unclear or high risk of bias in this domain due to insufficient reporting or open-label designs. Outcome assessment was generally considered at low risk of bias, particularly for objective measures such as axial length, refraction, or macular pigment optical density, whereas subjective outcomes (e.g., visual fatigue questionnaires) contributed to some concerns in a minority of studies. Attrition bias was low overall, although a few trials reported incomplete outcome data or dropouts without detailed handling strategies. Selective reporting was uncommon, as most studies reported prespecified outcomes consistent with their methods sections. No study was excluded from the review solely on the basis of risk of bias, but the identified methodological limitations were considered when interpreting the results.

### 3.4. Outcomes

#### 3.4.1. Effects of Carotenoids

Carotenoids, a class of dietary xanthophylls and related compounds with antioxidant and blue-light filtering properties, were the most extensively investigated nutritional supplements among the included trials. Five RCTs assessed the efficacy of astaxanthin, lutein, zeaxanthin, crocetin, or *Lycium barbarum* extracts enriched in zeaxanthin and lutein, spanning diverse populations from pediatric cohorts with active myopia progression to adults with high myopia or digital eye strain. Collectively, these studies provide convergent but heterogeneous evidence supporting a role of carotenoids in modulating ocular structure, function, and visual fatigue.

Astaxanthin was evaluated in a multicenter trial by Hecht et al. [24] involving 64 school-aged children (10–14 years) with mild-to-moderate computer vision syndrome (CVS). After 84 days of astaxanthin supplementation, significant improvements were observed in visual fatigue–related outcomes and stereopsis compared with placebo, whereas visual acuity, refraction, and accommodative parameters did not differ between groups. No adverse events were reported.

Lutein and zeaxanthin were the focus of two RCTs with distinct populations. Tanito et al. [30] randomized 22 healthy Japanese adults to receive either lutein (10 mg/day) or zeaxanthin (10 mg/day) for three months. Macular pigment optical density (MPOD) was quantified using resonance Raman spectrophotometry (RRS) and autofluorescence imaging (AFI). Lutein supplementation significantly increased MPOD by more than 20% at two and three months, particularly in individuals without high myopia. In contrast, zeaxanthin supplementation failed to yield consistent MPOD gains, irrespective of refractive status. Complementing this, Yoshida et al. [31] studied 44 highly myopic adults (axial length > 26 mm), of whom 28 completed six months of lutein supplementation at a higher dose (20 mg/day). In the overall cohort, MPOD changes were not statistically significant between groups; however, subgroup analysis revealed that eyes with axial length below 28.25 mm exhibited significant increases in MPOD (0.71 ± 0.20 to 0.78 ± 0.22, *p* = 0.02). Taken together, these studies indicate that lutein supplementation can augment macular pigment density, although this effect appears attenuated in the context of advanced axial elongation.

Crocetin, a saffron-derived apocarotenoid, was assessed in a multicenter Japanese RCT by Mori et al. [28] in 69 children aged 6–12 years with moderate myopia. Over 24 weeks, the crocetin group exhibited significantly less axial elongation (0.18 ± 0.02 mm vs. 0.21 ± 0.02 mm in placebo, *p* = 0.046) and a smaller myopic shift in spherical equivalent refraction (−0.33 ± 0.05 D vs. −0.41 ± 0.05 D, *p* = 0.049). No safety concerns were identified. Zeaxanthin from *Lycium barbarum* was examined by Zhang et al. [32] in 54 patients with high myopia categorized into severity grades 1–3. Participants were randomized to receive low-dose or high-dose *Lycium barbarum* (containing 10–20 mg of zeaxanthin and 1–2 mg of lutein), matched lutein controls, or placebo for three months. MPOD increased significantly in the high-dose *Lycium barbarum* group compared to high-dose lutein (*p* = 0.0403), whereas no difference was observed between low-dose conditions. MPOD correlated negatively with axial length and myopia severity, and positively with best-corrected visual acuity. These results highlight the potential superiority of zeaxanthin-rich interventions over lutein in preserving macular pigment in advanced myopia.

When considered collectively, the carotenoid trials demonstrate consistent trends toward improved retinal antioxidant status and functional outcomes, albeit with variability in the magnitude and context of effect. Pediatric data [24,28] suggest that carotenoids such as astaxanthin and crocetin can influence both symptom burden and structural progression, respectively, in early-stage populations. Adult studies [30,31,32] indicate that lutein and zeaxanthin supplementation enhances macular pigment density, though efficacy is moderated by axial elongation and disease stage. The heterogeneity of dosing regimens (4–20 mg/day), follow-up durations (3–24 weeks), and measurement techniques complicates direct comparisons. Nevertheless, the convergence of findings across trials underscores the plausible role of carotenoids as adjunctive strategies for myopia management and digital eye strain mitigation.

Overall, evidence from carotenoid trials indicates more consistent effects on macular pigment optical density and subjective or functional visual outcomes than on refractive error or axial elongation, with structural benefits observed in only a limited number of pediatric studies.

#### 3.4.2. Effects of Anthocyanins and Bilberry Derivatives

Anthocyanins, a class of flavonoid pigments abundant in bilberry (*Vaccinium myrtillus*) and related extracts, have long been proposed to enhance night vision and alleviate visual fatigue through antioxidant activity, vascular stabilization, and potential effects on photoreceptor physiology. Three randomized controlled trials spanning from the 1980s to the 2010s investigated purified anthocyanosides, cyaninoside chloride, and fermented bilberry extracts in myopic populations with or without night vision complaints. Collectively, these studies suggest that anthocyanin supplementation can improve mesopic contrast sensitivity, scotopic visual function, and patient-reported symptoms of asthenopia, though variability exists in the formulation, dosage, and methodological approaches employed.

Early clinical evidence was provided by Solé et al. [29], who conducted a controlled trial in 31 outpatients with myopia and night blindness. Participants were randomized to receive cyaninoside chloride, Heleniene, or no treatment, with outcomes assessed through photopic and mesopic acuity, electro-oculography, and adapto-electroretinography. Both agents improved photopic acuity (*p* < 0.05), but only cyaninoside chloride yielded significant benefits in mesopic and scotopic function (*p* < 0.01), as well as faster adaptation responses.

More rigorous methodology was applied in a double-blind, placebo-controlled RCT by Lee et al. [26], which enrolled 60 adults with low-to-moderate myopia and asthenopia. Participants received either purified high-dose anthocyanoside oligomers (100 mg twice daily, 85% purity) or placebo for four weeks. The anthocyanoside group reported symptom improvement in 73.3% of cases compared with only 3.3% in the placebo group (*p* < 0.0001). Objectively, contrast sensitivity under mesopic conditions improved significantly across all spatial frequencies in the active group, while no gains were observed with placebo. Importantly, no treatment-related adverse events were reported. This trial not only confirmed subjective benefits but also provided robust psychophysical evidence of enhanced contrast sensitivity, marking a significant advance over prior studies that often relied solely on self-reported outcomes or heterogeneous bilberry preparations of lower purity.

Extending this line of inquiry, Kamiya et al. [25] evaluated a fermented bilberry extract in a prospective, randomized, placebo-controlled study of myopic patients. The intervention was designed to maximize anthocyanin bioavailability through fermentation, a process shown to enhance gastrointestinal absorption. Patients receiving the fermented extract demonstrated favorable changes in visual function metrics relative to placebo, consistent with the hypothesis that anthocyanins modulate visual performance through both antioxidant and microvascular mechanisms. Although sample sizes were modest, the study provided contemporary evidence that anthocyanin supplementation can yield measurable functional benefits even in highly screened modern cohorts.

Taken together, anthocyanin-based interventions demonstrated consistent improvements in mesopic contrast sensitivity and subjective symptoms of asthenopia, while no evidence was available regarding effects on refractive progression or axial length.

#### 3.4.3. Effects of Combined Formulations

The only randomized controlled trial in this category was conducted by Lin et al. [27] in Taiwan. This double-blind, placebo-controlled study enrolled 44 young adults (20–31 years) with myopia and intensive digital device use. Participants received chewable tablets designed for sublingual–buccal absorption, containing blackcurrant and red grape extracts together with lutein, β-carotene, and zeaxanthin. This administration route was intended to enhance bioavailability by bypassing first-pass metabolism.

After eight weeks, the supplementation group showed significant improvements in binocular accommodative facility (BAF), increasing from 15.6 ± 4.2 to 18.1 ± 3.9 cycles per minute (*p* < 0.05), whereas the placebo group experienced a slight decline (−0.7 ± 3.2). The proportion of participants with “very poor” BAF (≤10 cpm) decreased by 25% in the intervention arm, while the proportion achieving regular accommodative performance (>15 cpm) increased. These findings suggest that combined supplementation enhances binocular function and mitigates visual fatigue associated with sustained near work.

Complementary in vitro experiments in ARPE-19 retinal pigment epithelial cells demonstrated protective effects against UVB and blue light exposure. The formulation increased cell viability (+13.8% relative to damaged controls), reduced reactive oxygen species generation by 77.6%, and downregulated pro-inflammatory gene expression (e.g., VEGFA, IL-1β).

Although limited by a modest sample size and short duration, this trial highlights two key points: first, combined formulations may exert greater benefits than single compounds; and second, sublingual–buccal delivery is a promising route to optimize the efficacy of nutraceuticals for ocular health. Together, these results suggest that multimodal supplementation could serve as an adjunct strategy for reducing visual fatigue and supporting accommodative function in myopic individuals or those at risk of progression.

Overall, evidence from combined formulations suggests potential synergistic benefits on accommodative function and visual fatigue, although conclusions are limited by the availability of a single short-term randomized trial.

## 4. Discussion

This systematic review demonstrates that nutritional supplementation, particularly with carotenoids, anthocyanins, and certain combined nutraceutical formulations, can produce modest but measurable effects on ocular parameters associated with myopia. Observed benefits include increases in macular pigment optical density, improvements in subjective visual performance, and, in certain pediatric populations, reductions in axial elongation. However, the evidence remains limited by small sample sizes, short intervention durations, and heterogeneity in both interventions and outcomes measures. Overall, effect sizes are generally modest when compared with established pharmacological and optical strategies for myopia control.

Our findings align with the hypothesis that oxidative stress, inflammation, and altered extracellular matrix remodeling contribute to myopia pathogenesis, and suggest that nutritional agents targeting these pathways may serve as adjunctive tools in prevention or control [33,34,35,36]. Notably, the most consistent benefits were observed for subjective symptoms of visual fatigue and macular pigment density, rather than direct measures of refractive progression. This pattern reflects the broader literature and highlights the challenge of translating promising mechanistic or biochemical effects into clinically robust outcomes.

This consistency likely reflects that both clinical trials and experimental research converge on the idea that nutrition influences biochemical pathways relevant to myopia, such as oxidative stress and inflammation [37]. However, the translation from mechanistic improvements (e.g., antioxidant activity) to clinically significant refractive changes is inherently challenging, particularly when most available trials are limited in scale and duration. The results of this review align with recent experimental studies supporting the role of micronutrients in myopia modulation. For example, Liang et al. [38] and Jiao et al. [12] demonstrated that exogenous vitamin D analogues can inhibit myopia progression in animal models by maintaining choroidal and scleral integrity and enhancing collagen synthesis via VDR-dependent signaling. Although none of the included RCTs directly evaluated vitamin D supplementation in humans, these experimental findings provide a mechanistic rationale for future trials. Observational studies in humans, such as Yazar et al. [39], have reported lower serum 25(OH)D levels in myopic individuals compared to non-myopes, reinforcing the plausibility of a role for vitamin D, although causality remains unproven and may be confounded by sun exposure and lifestyle. The agreement between animal studies and human observational data may be explained by shared biological mechanisms involving vitamin D pathways. However, the lack of direct human RCTs and the possibility of confounding factors, such as sunlight exposure or outdoor activity, underscore why these findings, although suggestive, cannot yet be considered definitive evidence for supplementation in clinical practice.

Similarly, biomarker studies show that high myopia is associated with lower plasma levels of retinol (vitamin A) and α-tocopherol (vitamin E) and higher oxidative stress [10]. Our review confirms that supplementation with carotenoids or anthocyanin-rich extracts can improve functional outcomes, such as contrast sensitivity and visual fatigue, especially in high-demand visual environments. However, large-scale Mendelian randomization analyses [15] do not support a direct causal relationship between vitamin A supplementation and myopia risk, highlighting the complexity of nutrient–phenotype relationships and the potential influence of confounding factors. These contrasting findings underscore the importance of study design: while biomarker and supplementation studies may reveal associations or short-term improvements, Mendelian randomization offers a more robust approach to inferring causality. The lack of a causal signal may reflect unmeasured confounding in observational data, genetic differences in metabolism, or the multifactorial nature of myopia development.

Our findings differ somewhat from several large cross-sectional and cohort studies, which have reported null associations between dietary intake of carotenoids, vitamins, or macronutrients and myopia prevalence or progression. For example, analyses of the NHANES cohort [40,41] and the GUSTO birth cohort [42] found no significant link between intake of lutein, zeaxanthin, vitamin D, or macronutrients and refractive outcomes in children or adolescents. Similarly, Burke et al. [43] reported no association between dietary zinc and myopia in US adolescents. This apparent discordance likely reflects differences in study design (RCT vs. observational), measurement of nutritional status (dietary intake vs. supplementation vs. plasma levels), bioavailability, and population genetics. Such discrepancies can be attributed to differences in methodological approaches: randomized controlled trials offer higher internal validity but often study narrower, more selected populations, while observational studies cover larger, more heterogeneous groups but are more prone to confounding and measurement error. Additionally, differences in nutrient bioavailability, adherence, and genetic background may also play a role in the divergent findings. On the other hand, emerging evidence suggests a more consistent relationship between PUFAs, particularly omega-3s, and myopia risk. Several recent studies [44,45] have found that higher plasma or dietary levels of omega-3 PUFAs are associated with lower myopia risk and shorter axial length in children and adolescents. Although our review did not identify any RCTs evaluating omega-3 supplementation, these findings suggest a potentially promising avenue for future interventional research. This consistency may be due to the specific biological roles of omega-3 PUFAs in retinal and choroidal health, as well as the fact that both experimental and epidemiological evidence point towards a genuine protective effect. The lack of RCTs, however, highlights the need for well-designed interventional studies to confirm whether these associations translate into real clinical benefit.

Collectively, our synthesis and the referenced literature point toward plausible mechanisms through which nutritional supplementation may influence ocular growth. Carotenoids and anthocyanins can reduce oxidative stress and modulate retinal inflammatory pathways, while vitamin D and its analogues have been shown to influence scleral remodeling and collagen synthesis. PUFAs may enhance choroidal perfusion and alter retinal signaling relevant to ocular growth. Despite these mechanistic insights, their translation into meaningful clinical benefit remains to be fully demonstrated in large, long-term trials.

A key strength of this review is its rigorous methodology: the systematic identification and selection of RCTs, independent risk of bias assessment, and focus on clinically relevant outcomes. By including only randomized trials, this synthesis provides a more robust appraisal of the true efficacy of nutritional interventions, minimizing confounding and selection bias common in observational studies. Furthermore, the inclusion of trials from diverse geographic regions and with varying study populations enhances the generalizability of the findings within the limits of the available evidence.

Nevertheless, several important limitations must be acknowledged. First, the number of eligible RCTs is limited, these nine studies represent the entirety of the available randomized controlled trial evidence to date, as our comprehensive search identified no additional qualifying trials. Sample sizes were generally small, and follow-up periods were short, limiting the ability to assess long-term effects on refractive progression or incident myopia. The heterogeneity across trials in terms of intervention composition, dosing, measurement endpoints, and participant characteristics further complicates direct comparison and precludes meaningful quantitative synthesis. Additionally, most studies focused on surrogate outcomes rather than objective changes in refractive error or axial length, restricting the clinical significance of the observed effects. Another limitation concerns external validity, as the majority of included studies were conducted in East Asian populations, where baseline risk factors for myopia and dietary patterns may differ from those in Western cohorts. The lack of studies targeting key subgroups, such as young children at highest risk of progression, populations with nutritional deficiencies, or those with strong familial risk, further limits the applicability of these findings. There is also potential for publication bias, as positive trials are more likely to be published and some studies received industry support. An exploratory funnel plot was generated for transparency; however, its interpretation is limited by the small number of trials and substantial heterogeneity. Therefore, residual bias cannot be excluded. Although risk-of-bias assessments were conducted rigorously, they cannot fully eliminate concerns, particularly in small-scale or open-label trials. A formal certainty-of-evidence framework (e.g., GRADE) was not applied due to the absence of pooled effect estimates and the exploratory, non–meta-analytic nature of the available RCT evidence.

Additional methodological heterogeneity should be considered when interpreting these findings. Across trials assessing macular pigment optical density, different measurement techniques were used, including heterochromatic flicker photometry, resonance Raman spectrophotometry, and autofluorescence-based methods, which may differ in sensitivity and comparability. Furthermore, dose levels and formulations varied substantially between studies, limiting direct dose–response comparisons. Finally, although compliance was generally reported, it was assessed using different approaches and not systematically compared across trials, introducing additional uncertainty in the interpretation of treatment effects. Future research should prioritize large-scale, multicenter RCTs with standardized intervention protocols and longer follow-up, ideally incorporating both clinical endpoints and mechanistic biomarkers such as serum or plasma nutrient levels, oxidative stress markers, and imaging of scleral and choroidal structure. Special attention should be given to evaluating omega-3 supplementation, vitamin D analogues, and synergistic combinations of nutraceuticals, as well as to identifying which subgroups might benefit most from such interventions. Integration of genetic and environmental data, along with careful control for confounders such as outdoor exposure, near work, and baseline nutritional status, will be essential to clarify the role of nutrition in myopia prevention and management.

## 5. Conclusions

In conclusion, randomized controlled trials suggest that certain nutritional supplements, particularly carotenoids, anthocyanins, and possibly omega-3 polyunsaturated fatty acids, may offer modest benefits for visual function, macular pigment density, and subjective visual comfort in individuals with myopia. However, current evidence does not support a clinically meaningful effect on slowing myopia progression or reducing axial elongation. The variability of findings across studies, combined with the disconnect between interventional trials and large-scale epidemiological data, suggests that nutritional supplementation should be considered as an adjunctive measure rather than a primary approach for myopia management.

Future research should focus on large, rigorously designed randomized trials with extended follow-up and standardized outcome measures, including mechanistic biomarkers, to better define the role of nutrition in myopia care and to identify subgroups most likely to benefit. Until such evidence emerges, routine use of nutritional supplements for myopia prevention or treatment cannot be generally recommended, although it may be appropriate for individuals with documented nutritional deficiencies or specific visual demands.

Ultimately, a comprehensive understanding of how nutrition interacts with genetic predisposition and environmental exposures will be crucial for developing more effective, evidence-based strategies for the prevention and control of myopia.

## Figures and Tables

**Table 1:** PICOS framework for study eligibility.

Component	Description
Population	Individuals with myopia or at risk of developing myopia (children and adults)
Intervention	Nutritional supplementation (carotenoids, vitamins, anthocyanins, polyunsaturated fatty acids, and combined formulations)
Comparator	Placebo or alternative nutritional formulations
Outcomes	Primary: refractive error, axial length, macular pigment optical density. Secondary: visual acuity, contrast sensitivity, accommodative function, visual fatigue
Study design	Randomized controlled trials

**Table 2:** Baseline characteristics of the 9 included studies.

Author (Year)	Country	Study Design	Sample Size	Mean Age (Years)	Primary Outcome	Treatment/Modality	Follow-Up	Measurement Method	COI
Hecht et al. (2025) [24]	India (multicenter)	RCT	64 (32 astaxanthin, 32 placebo)	11.7 ± 1.3 (range 10–14)	Change in computer vision syndrome symptoms and visual fatigue (CVS-Q and Visual Fatigue Likert Scale scores)	Astaxanthin 4 mg/day (AstaReal^®,^ (Osaka, Japan) vs. placebo (84 days)	14, 28, 56, and 84 days	CVS-Q, Visual Fatigue Likert Scale (VFLS), stereopsis (TNO test), Schirmer I test, pupil size, VA, refraction, NPA, near exophoria, blinking	No
Kamiya et al. (2013) [25]	Japan	RCT	30 eyes of 30 myopic adults	39.5 ± 7.2 (range 31–53)	Change in mesopic contrast sensitivity and accommodative function	Fermented bilberry extract 400 mg/day (Ajinomoto Co., Inc.; Chūō, Tokio) vs. placebo, oral, 4 weeks	4 weeks treatment, 4 weeks washout, 4 weeks cross-over	Visual acuity (logMAR), refraction, pupil constriction (TrilRIS), amplitude of accommodation (D’ACOMO accommodometer; Milan, Italy)), mesopic contrast sensitivity (VCTS-6500, AULCSF)	No
Lee et al. (2005) [26]	South Korea	RCT	60 (30 anthocyanoside, 30 placebo)	Anthocyanoside: 41.1 ± 13.1; Placebo: 36.0 ± 12.6 (range 18–65)	Change in subjective asthenopia symptoms and mesopic contrast sensitivity	Purified high-dose anthocyanoside oligomer (Eyezone^®^, 100 mg tablet, 85% anthocyanosides) twice daily for 4 weeks vs. placebo	4 weeks	Symptom questionnaire (asthenopia), mesopic contrast sensitivity with morphoscopic CS (Visual Capacity Analyzer, ACV)	Yes
Lin et al. (2025) [27]	Taiwan	RCT	44 (24 intervention, 20 placebo)	21.8 ± 1.6 (intervention), 22.4 ± 2.1 (placebo)	Change in binocular accommodative facility	Chewable tablet (sublingual-buccal absorption): blackcurrant, red grape, lutein, β-carotene, zeaxanthin; 2 tablets/day for 8 weeks	Baseline and 8 weeks	Binocular accommodative facility (flipper ±2.00 D), amplitude of accommodation (RAF ruler)	Yes
Mori et al. (2019) [28]	Japan	RCT	69 (39 crocetin, 30 placebo, 67 completed)	10.2 ± 1.3 (range 6–12)	Change in axial length (primary) and cycloplegic spherical equivalent refraction	Crocetin 7.5 mg/day vs. placebo, oral capsules	24 weeks (baseline, 4, 12, 24 weeks)	Cycloplegic spherical equivalent refraction (auto-refractometer, NIDEK ARK-730A), axial length (IOLMaster 700), OCT for choroidal thickness	No
Solé et al. (1984) [29]	France	RCT	31 outpatients (myopia and night blindness)	NR	Change in mesopic and scotopic visual function (visual acuity and electrophysiological adaptation responses)	Cyaninoside chloride vs. Heleniene vs. control	NR (short-term; duration not specified)	Photopic & mesopic visual acuity, electro-oculography, adapto-electroretinography	No
Tanito et al. (2012) [30]	Japan	RCT	22 (11 lutein, 11 zeaxanthin)	39.6 ± 2.1 (lutein), 38.3 ± 3.2 (zeaxanthin)	Change in macular pigment optical density (MPOD)	Lutein 10 mg/day vs. Zeaxanthin 10 mg/day (3 months)	1, 2, and 3 months	Resonance Raman spectrophotometry (RRS), autofluorescence imaging (AFI)	No
Yoshida et al. (2023) [31]	Japan	RCT	28 eyes (15 lutein, 13 placebo, originally 44 enrolled)	Lutein: 46.5 ± 3.5; Control: 42.8 ± 6.6	Change in macular pigment optical density (MPOD)	Lutein 20 mg/day (oral) vs. placebo for 6 months	Baseline, 3, 6 months	MPOD (heterochromatic flicker photometry), BCVA (logMAR, Nidek SC-1600), contrast sensitivity (CSV-1000E, 3–18 cpd), full-field ERG	No
Zhang et al. (2022) [32]	China	RCT	96 eyes of 54 patients (categories 1–3 high myopia)	46.3 ± 12.3 (range 22–72)	Change in macular pigment optical density (MPOD)	Low-dose *Lycium barbarum* (10 g; 10 mg zeaxanthin + 1 mg lutein); high-dose *Lycium barbarum* (20 g; 20 mg zeaxanthin + 2 mg lutein); matched lutein controls (1 mg or 2 mg); blank control	3 months	MPOD by heterochromatic flicker photometry (MPSII^®^), BCVA (logMAR), AL, IOP, OCT	No

## Data Availability

The original contributions presented in this study are included in the article/Supplementary Materials. Further inquiries can be directed to the corresponding author.

## Supplementary Materials

The following supporting information can be downloaded at: https://www.mdpi.com/article/10.3390/nu18010004/s1, Supplementary File S1: PRISMA 2020 checklist; Supplementary File S2: Full electronic database search strategies (PubMed, Web of Science, Scopus); Supplementary File S3: Baseline characteristics of the 9 included studies. Supplementary File S4: Funnel Plot of Included RCTs.
