# Supplementary material for: Nutritional Supplementation for Myopia Prevention and Control: A Systematic Review of Randomized Controlled Trials
Source: Nutrients. 2025 Dec 19;18(1):4. doi: 10.3390/nu18010004 (PMC12787848; doi:10.3390/nu18010004)
Supplement: Supplementary file 1 [file nutrients-18-00004-s001.zip › nutrients-4049080-supplementary/Additional file S2.pdf]

Additional File S2. Full electronic search strategies.

<u>Pubmed</u>

Search: **("myopia"[MeSH Terms] OR myopia OR myopic OR "spherical equivalent" OR "axial length" OR "axial elongation") AND ("diet"[MeSH Terms] OR diet OR "nutrition" OR nutrition OR dietary OR food OR eating OR nutrient* OR micronutrient* OR supplement* OR supplementation OR vitamin* OR antioxidant* OR lutein OR zeaxanthin OR carotenoid* OR anthocyanin* OR anthocyanoside* OR bilberry OR "Lycium barbarum" OR goji OR nutraceutical* OR "functional food*" OR "omega-3 fatty acid*" OR "omega-6 fatty acid*" OR "fatty acid*" OR retinol OR "vitamin D" OR "vitamin A" OR "vitamin E" OR "vitamin C" OR zinc OR calcium OR magnesium OR selenium OR copper OR iron OR herbal OR phytotherapy OR "plant extract*" OR "traditional medicine" OR "Chinese medicine") NOT (orthokeratology OR "contact lens*" OR spectacle* OR lens OR atropine OR pirenzepine OR "eye drop*" OR intravitreal OR bevacizumab OR ranibizumab OR aflibercept OR anti-VEGF OR laser OR surgery OR surgical OR keratoplasty OR keratotomy OR LASIK OR LASEK OR PRK OR "refractive surgery" OR cataract OR glaucoma)**

(("myopia"[MeSH Terms] OR ("myopia"[MeSH Terms] OR "myopia"[All Fields] OR "myopias"[All Fields]) OR ("myopia"[MeSH Terms] OR "myopia"[All Fields] OR "myopic"[All Fields] OR "myopics"[All Fields]) OR "spherical equivalent"[All Fields] OR "axial length"[All Fields] OR "axial elongation"[All Fields]) AND ("diet"[MeSH Terms] OR ("diet"[MeSH Terms] OR "diet"[All Fields]) OR "nutrition"[All Fields] OR ("nutrition s"[All Fields] OR "nutritional status"[MeSH Terms] OR ("nutritional"[All Fields] AND "status"[All Fields]) OR "nutritional status"[All Fields] OR "nutrition"[All Fields] OR "nutritional sciences"[MeSH Terms] OR ("nutritional"[All Fields] AND "sciences"[All Fields]) OR "nutritional sciences"[All Fields] OR "nutritional"[All Fields] OR "nutritionals"[All Fields] OR "nutritions"[All Fields] OR "nutritive"[All Fields]) OR ("diet"[MeSH Terms] OR "diet"[All Fields] OR "dietary"[All Fields] OR "dietaries"[All Fields]) OR ("food"[MeSH Terms] OR "food"[All Fields]) OR ("eating"[MeSH Terms] OR "eating"[All Fields]) OR "nutrient*"[All Fields] OR "micronutrient*"[All Fields] OR "supplement*"[All Fields] OR ("supplemental"[All Fields] OR "supplementating"[All Fields] OR "supplementation"[All Fields] OR "supplementation s"[All Fields] OR "supplementations"[All Fields] OR "supplemention"[All Fields]) OR "vitamin*"[All Fields] OR "antioxidant*"[All Fields] OR ("lutein"[Supplementary Concept] OR "lutein"[All Fields] OR "lutein"[MeSH Terms] OR "lutein s"[All Fields] OR "luteins"[All Fields]) OR ("zeaxanthine"[All Fields] OR "zeaxanthins"[Supplementary Concept] OR "zeaxanthins"[All Fields] OR "zeaxanthin"[All Fields] OR "zeaxanthins"[MeSH Terms]) OR "carotenoid*"[All Fields] OR "anthocyanin*"[All Fields] OR "anthocyanoside*"[All Fields] OR ("vaccinium myrtillus extract"[Supplementary Concept] OR "vaccinium myrtillus extract"[All Fields] OR "bilberry"[All Fields] OR "vaccinium myrtillus"[MeSH Terms] OR ("vaccinium"[All Fields] AND "myrtillus"[All Fields]) OR "vaccinium myrtillus"[All Fields] OR "bilberries"[All Fields]) OR "Lycium barbarum"[All Fields] OR "goji"[All Fields] OR "nutraceutical*"[All Fields] OR "functional food*"[All Fields] OR "omega 3 fatty acid*"[All Fields] OR "omega 6 fatty acid*"[All Fields] OR "fatty acid*"[All Fields] OR ("vitamin A"[Supplementary Concept] OR "vitamin A"[All Fields] OR "retinol"[All Fields] OR "vitamin A"[MeSH Terms] OR "retinols"[All Fields]) OR "vitamin D"[All Fields] OR "vitamin A"[All Fields] OR "vitamin E"[All Fields] OR "vitamin C"[All Fields] OR ("zinc"[Supplementary Concept] OR "zinc"[All Fields] OR "zinc"[MeSH Terms]) OR ("calcium"[Supplementary Concept] OR "calcium"[All Fields] OR "calcium"[MeSH Terms] OR "calciums"[All Fields] OR "calcium s"[All Fields]) OR

("magnesium"[Supplementary Concept] OR "magnesium"[All Fields] OR "magnesium"[MeSH Terms] OR "magnesium s"[All Fields] OR "magnesiums"[All Fields]) OR ("selenium"[Supplementary Concept] OR "selenium"[All Fields] OR "selenium"[MeSH Terms] OR "selenium s"[All Fields] OR "seleniums"[All Fields]) OR ("copper"[Supplementary Concept] OR "copper"[All Fields] OR "copper"[MeSH Terms] OR "coppers"[All Fields] OR "copper s"[All Fields]) OR ("iron"[Supplementary Concept] OR "iron"[All Fields] OR "iron"[MeSH Terms]) OR ("herbal medicine"[MeSH Terms] OR ("herbal"[All Fields] AND "medicine"[All Fields]) OR "herbal medicine"[All Fields] OR "herbalism"[All Fields] OR "herbal"[All Fields] OR "herbals"[All Fields]) OR ("phytotherapy"[MeSH Terms] OR "phytotherapy"[All Fields] OR "phytotherapies"[All Fields]) OR "plant extract*"[All Fields] OR "traditional medicine"[All Fields] OR "Chinese medicine"[All Fields])) NOT ("orthokeratology"[All Fields] OR "contact lens*"[All Fields] OR "spectacle*"[All Fields] OR ("lenses"[MeSH Terms] OR "lenses"[All Fields] OR "lens"[All Fields] OR "lens, crystalline"[MeSH Terms] OR ("lens"[All Fields] AND "crystalline"[All Fields]) OR "crystalline lens"[All Fields]) OR ("atropine"[Supplementary Concept] OR "atropine"[All Fields] OR "atropin"[All Fields] OR "atropine"[MeSH Terms] OR "atropinization"[All Fields] OR "atropinized"[All Fields] OR "hyoscyamine"[Supplementary Concept] OR "hyoscyamine"[All Fields] OR "hyoscyamine"[MeSH Terms]) OR ("pirenzepine"[Supplementary Concept] OR "pirenzepine"[All Fields] OR "pirenzepin"[All Fields] OR "pirenzepine"[MeSH Terms]) OR "eye drop*"[All Fields] OR ("intravitral"[All Fields] OR "intravitreal"[All Fields] OR "intravitreally"[All Fields] OR "intravitreous"[All Fields] OR "intravitreously"[All Fields]) OR ("bevacizumab"[Supplementary Concept] OR "bevacizumab"[All Fields] OR "bevacizumab"[MeSH Terms] OR "bevacizumab s"[All Fields]) OR ("ranibizumab"[Supplementary Concept] OR "ranibizumab"[All Fields] OR "ranibizumab"[MeSH Terms]) OR ("aflibercept"[Supplementary Concept] OR "aflibercept"[All Fields]) OR "anti-VEGF"[All Fields] OR ("laser s"[All Fields] OR "lasers"[MeSH Terms] OR "lasers"[All Fields] OR "laser"[All Fields] OR "lasered"[All Fields] OR "lasering"[All Fields]) OR ("surgery"[MeSH Subheading] OR "surgery"[All Fields] OR "surgical procedures, operative"[MeSH Terms] OR ("surgical"[All Fields] AND "procedures"[All Fields] AND "operative"[All Fields]) OR "operative surgical procedures"[All Fields] OR "general surgery"[MeSH Terms] OR ("general"[All Fields] AND "surgery"[All Fields]) OR "general surgery"[All Fields] OR "surgery s"[All Fields] OR "surgerys"[All Fields] OR "surgeries"[All Fields]) OR ("surgical procedures, operative"[MeSH Terms] OR ("surgical"[All Fields] AND "procedures"[All Fields] AND "operative"[All Fields]) OR "operative surgical procedures"[All Fields] OR "surgical"[All Fields] OR "surgically"[All Fields] OR "surgicals"[All Fields]) OR ("corneal transplantation"[MeSH Terms] OR ("corneal"[All Fields] AND "transplantation"[All Fields]) OR "corneal transplantation"[All Fields] OR "keratoplasties"[All Fields] OR "keratoplasty"[All Fields]) OR ("keratotomies"[All Fields] OR "keratotomy"[All Fields]) OR ("keratomileusis, laser in situ"[MeSH Terms] OR ("keratomileusis"[All Fields] AND "laser"[All Fields] AND "situ"[All Fields]) OR "laser in situ keratomileusis"[All Fields] OR "lasik"[All Fields]) OR ("keratectomy, subepithelial, laser assisted"[MeSH Terms] OR ("keratectomy"[All Fields] AND "subepithelial"[All Fields] AND "laser assisted"[All Fields]) OR "laser-assisted subepithelial keratectomy"[All Fields] OR "lasek"[All Fields]) OR "PRK"[All Fields] OR "refractive surgery"[All Fields] OR ("cataract"[MeSH Terms] OR "cataract"[All Fields] OR "cataracts"[All Fields] OR "cataractic"[All Fields] OR "cataractous"[All Fields]) OR ("glaucoma"[MeSH Terms] OR "glaucoma"[All Fields] OR "glaucomas"[All Fields] OR "glaucoma s"[All Fields]))

Translations

myopia: "myopia"[MeSH Terms] OR "myopia"[All Fields] OR "myopias"[All Fields]

myopic: "myopia"[MeSH Terms] OR "myopia"[All Fields] OR "myopic"[All Fields] OR "myopics"[All Fields]
diet: "diet"[MeSH Terms] OR "diet"[All Fields]
nutrition: "nutrition's"[All Fields] OR "nutritional status"[MeSH Terms] OR ("nutritional"[All Fields] AND "status"[All Fields]) OR "nutritional status"[All Fields] OR "nutrition"[All Fields] OR "nutritional sciences"[MeSH Terms] OR ("nutritional"[All Fields] AND "sciences"[All Fields]) OR "nutritional sciences"[All Fields] OR "nutritional"[All Fields] OR "nutritionals"[All Fields] OR "nutritions"[All Fields] OR "nutritive"[All Fields]
dietary: "diet"[MeSH Terms] OR "diet"[All Fields] OR "dietary"[All Fields] OR "dietaries"[All Fields]
food: "food"[MeSH Terms] OR "food"[All Fields]
eating: "eating"[MeSH Terms] OR "eating"[All Fields]
supplementation: "supplemental"[All Fields] OR "supplementating"[All Fields] OR "supplementation"[All Fields] OR "supplementation's"[All Fields] OR "supplementations"[All Fields] OR "supplemention"[All Fields]
lutein: "lutein"[Supplementary Concept] OR "lutein"[All Fields] OR "lutein"[MeSH Terms] OR "lutein's"[All Fields] OR "luteins"[All Fields]
zeaxanthin: "zeaxanthine"[All Fields] OR "zeaxanthins"[Supplementary Concept] OR "zeaxanthins"[All Fields] OR "zeaxanthin"[All Fields] OR "zeaxanthins"[MeSH Terms]
bilberry: "vaccinium myrtillus extract"[Supplementary Concept] OR "vaccinium myrtillus extract"[All Fields] OR "bilberry"[All Fields] OR "vaccinium myrtillus"[MeSH Terms] OR ("vaccinium"[All Fields] AND "myrtillus"[All Fields]) OR "vaccinium myrtillus"[All Fields] OR "bilberries"[All Fields]
retinol: "vitamin a"[Supplementary Concept] OR "vitamin a"[All Fields] OR "retinol"[All Fields] OR "vitamin a"[MeSH Terms] OR "retinols"[All Fields]
zinc: "zinc"[Supplementary Concept] OR "zinc"[All Fields] OR "zinc"[MeSH Terms]
calcium: "calcium"[Supplementary Concept] OR "calcium"[All Fields] OR "calcium"[MeSH Terms] OR "calciums"[All Fields] OR "calcium's"[All Fields]
magnesium: "magnesium"[Supplementary Concept] OR "magnesium"[All Fields] OR "magnesium"[MeSH Terms] OR "magnesium's"[All Fields] OR "magnesiums"[All Fields]
selenium: "selenium"[Supplementary Concept] OR "selenium"[All Fields] OR "selenium"[MeSH Terms] OR "selenium's"[All Fields] OR "seleniums"[All Fields]
copper: "copper"[Supplementary Concept] OR "copper"[All Fields] OR "copper"[MeSH Terms] OR "coppers"[All Fields] OR "copper's"[All Fields]
iron: "iron"[Supplementary Concept] OR "iron"[All Fields] OR "iron"[MeSH Terms]
herbal: "herbal medicine"[MeSH Terms] OR ("herbal"[All Fields] AND "medicine"[All Fields]) OR "herbal medicine"[All Fields] OR "herbalism"[All Fields] OR "herbal"[All Fields] OR "herbals"[All Fields]
phytotherapy: "phytotherapy"[MeSH Terms] OR "phytotherapy"[All Fields] OR "phytotherapies"[All Fields]
lens: "lenses"[MeSH Terms] OR "lenses"[All Fields] OR "lens"[All Fields] OR "lens, crystalline"[MeSH Terms] OR ("lens"[All Fields] AND "crystalline"[All Fields]) OR "crystalline lens"[All Fields]
atropine: "atropine"[Supplementary Concept] OR "atropine"[All Fields] OR "atropin"[All Fields] OR "atropine"[MeSH Terms] OR "atropinization"[All Fields] OR "atropinized"[All Fields] OR "hyoscyamine"[Supplementary Concept] OR "hyoscyamine"[All Fields] OR "hyoscyamine"[MeSH Terms]

pirenzepine: "pirenzepine"[Supplementary Concept] OR "pirenzepine"[All Fields] OR "pirenzepin"[All Fields] OR "pirenzepine"[MeSH Terms]
intravitreal: "intravitral"[All Fields] OR "intravitreal"[All Fields] OR "intravitreally"[All Fields] OR "intravitreous"[All Fields] OR "intravitreously"[All Fields]
bevacizumab: "bevacizumab"[Supplementary Concept] OR "bevacizumab"[All Fields] OR "bevacizumab"[MeSH Terms] OR "bevacizumab's"[All Fields]
ranibizumab: "ranibizumab"[Supplementary Concept] OR "ranibizumab"[All Fields] OR "ranibizumab"[MeSH Terms]
aflibercept: "aflibercept"[Supplementary Concept] OR "aflibercept"[All Fields]
laser: "laser's"[All Fields] OR "lasers"[MeSH Terms] OR "lasers"[All Fields] OR "laser"[All Fields] OR "lasered"[All Fields] OR "lasering"[All Fields]
surgery: "surgery"[Subheading] OR "surgery"[All Fields] OR "surgical procedures, operative"[MeSH Terms] OR ("surgical"[All Fields] AND "procedures"[All Fields] AND "operative"[All Fields]) OR "operative surgical procedures"[All Fields] OR "general surgery"[MeSH Terms] OR ("general"[All Fields] AND "surgery"[All Fields]) OR "general surgery"[All Fields] OR "surgery's"[All Fields] OR "surgerys"[All Fields] OR "surgeries"[All Fields]
surgical: "surgical procedures, operative"[MeSH Terms] OR ("surgical"[All Fields] AND "procedures"[All Fields] AND "operative"[All Fields]) OR "operative surgical procedures"[All Fields] OR "surgical"[All Fields] OR "surgically"[All Fields] OR "surgicals"[All Fields]
keratoplasty: "corneal transplantation"[MeSH Terms] OR ("corneal"[All Fields] AND "transplantation"[All Fields]) OR "corneal transplantation"[All Fields] OR "keratoplasties"[All Fields] OR "keratoplasty"[All Fields]
keratotomy: "keratotomies"[All Fields] OR "keratotomy"[All Fields]
LASIK: "keratomileusis, laser in situ"[MeSH Terms] OR ("keratomileusis"[All Fields] AND "laser"[All Fields] AND "situ"[All Fields]) OR "laser in situ keratomileusis"[All Fields] OR "lasik"[All Fields]
LASEK: "keratectomy, subepithelial, laser-assisted"[MeSH Terms] OR ("keratectomy"[All Fields] AND "subepithelial"[All Fields] AND "laser-assisted"[All Fields]) OR "laser-assisted subepithelial keratectomy"[All Fields] OR "lasek"[All Fields]
cataract: "cataract"[MeSH Terms] OR "cataract"[All Fields] OR "cataracts"[All Fields] OR "cataractic"[All Fields] OR "cataractous"[All Fields]
glaucoma: "glaucoma"[MeSH Terms] OR "glaucoma"[All Fields] OR "glaucomas"[All Fields] OR "glaucoma's"[All Fields]

<u>Web of Science</u>
TS=(
(myopia OR myopic OR "spherical equivalent" OR "axial length" OR "axial elongation")
AND
(diet OR nutrition OR dietary OR food OR eating
OR nutrient* OR micronutrient* OR supplement* OR supplementation
OR vitamin* OR antioxidant* OR lutein OR zeaxanthin OR carotenoid* OR anthocyanin* OR anthocyanoside*
OR bilberry OR "Lycium barbarum" OR goji OR nutraceutical* OR "functional food*"
OR "omega-3 fatty acid*" OR "omega-6 fatty acid*" OR "fatty acid*" OR retinol
OR "vitamin D" OR "vitamin A" OR "vitamin E" OR "vitamin C"
OR zinc OR calcium OR magnesium OR selenium OR copper OR iron

OR herbal OR phytotherapy OR "plant extract*" OR "traditional medicine" OR "Chinese medicine")
NOT
(orthokeratology OR "contact lens*" OR spectacle* OR lens
OR atropine OR pirenzepine OR "eye drop*" OR intravitreal
OR bevacizumab OR ranibizumab OR aflibercept OR anti-VEGF
OR laser OR surgery OR surgical OR keratoplasty OR keratotomy
OR LASIK OR LASEK OR PRK OR "refractive surgery" OR cataract OR glaucoma)
)

<u>Scopus</u>

TITLE-ABS-KEY(
(myopia OR myopic OR "spherical equivalent" OR "axial length" OR "axial elongation")
AND
(diet OR nutrition OR dietary OR food OR eating
OR nutrient* OR micronutrient* OR supplement* OR supplementation
OR vitamin* OR antioxidant* OR lutein OR zeaxanthin OR carotenoid* OR anthocyanin* OR anthocyanoside*
OR bilberry OR "Lycium barbarum" OR goji OR nutraceutical* OR "functional food*"
OR "omega-3 fatty acid*" OR "omega-6 fatty acid*" OR "fatty acid*" OR retinol
OR "vitamin D" OR "vitamin A" OR "vitamin E" OR "vitamin C"
OR zinc OR calcium OR magnesium OR selenium OR copper OR iron
OR herbal OR phytotherapy OR "plant extract*" OR "traditional medicine" OR "Chinese medicine")
AND NOT
(orthokeratology OR "contact lens*" OR spectacle* OR lens
OR atropine OR pirenzepine OR "eye drop*" OR intravitreal
OR bevacizumab OR ranibizumab OR aflibercept OR anti-VEGF
OR laser OR surgery OR surgical OR keratoplasty OR keratotomy
OR LASIK OR LASEK OR PRK OR "refractive surgery" OR cataract OR glaucoma)
)
