# Supplementary material for: Nutritional Supplementation for Myopia Prevention and Control: A Systematic Review of Randomized Controlled Trials
Source: Nutrients. 2025 Dec 19;18(1):4. doi: 10.3390/nu18010004 (PMC12787848; doi:10.3390/nu18010004)
Supplement: Supplementary file 1 [file nutrients-18-00004-s001.zip › nutrients-4049080-supplementary/Additional file S3.pdf]

Additional file S3. Risk of bias judgement.

Hecht et al. (2025)

A) Participants were randomized 1:1 using a stratified block randomization (block size = 4) performed with StatsDirect software, clearly described in the methods.

B) Astaxanthin and placebo capsules were identical vegetarian soft gels, both manufactured and provided by Fuji Chemical Industries. Group allocation was concealed from investigators, participants, and outcome assessors until the study was complete.

C) The study was double-blind. Both participants (children and parents) and study staff were unaware of treatment allocation. Capsules were indistinguishable.

D) Clinical assessments (CVS-Q, VFLS, stereopsis, pupil size, Schirmer I, etc.) were performed by masked investigators. The article explicitly states adherence to GCP and trial blinding procedures.

E) A total of 64 participants were randomized (32 per group) with 99% compliance and minimal attrition (LTFU not significant, well documented). No evidence of differential dropout between groups.

F) The trial was registered prospectively (ClinicalTrials.gov NCT05602402; CTRI/2022/10/046606). All primary and secondary outcomes described in the protocol (CVS-Q, VFLS, stereopsis, pupil size, tear production, safety) were reported in the results.

H) The study was funded and sponsored by Fuji Chemical Industries and AstaReal, the manufacturer of the tested supplement. Several authors are employees of AstaReal or Fuji Chemical. Although this was disclosed, potential industry sponsorship bias cannot be excluded.

Kamiya et al. (2013)

A) Participants were randomly assigned to one of two regimens (fermented bilberry extract vs placebo), with clear description of cross-over design and randomization by a designated study controller.

B) Active and placebo capsules were identical in appearance, with allocation controlled independently and concealed from investigators and participants.

C) The study was explicitly double-blind. Both volunteers and investigators were unaware of treatment sequence, and indistinguishable capsules ensured effective masking.

D) Visual outcomes (accommodation with D'ACOMO, mesopic contrast sensitivity with VCTS-6500, refraction, pupil constriction) were measured by a single experienced masked technician using standardized devices, minimizing detection bias.

E) All 30 participants completed both treatment periods. No dropouts or missing data were reported, and crossover compliance was complete.

F) The trial was registered in the UMIN Clinical Trials Registry (UMIN000007741). All prespecified outcomes (accommodation, contrast sensitivity, refraction, pupil function, VA) were reported. No evidence of unreported results.

H) The fermented bilberry extract product was commercially available in Japan and supplied by Ajinomoto Co., Inc. Potential industry involvement could introduce bias, although not explicitly declared as funding. The small sample size (n=30) and short duration (4 weeks per arm) also limit generalizability.

Lee et al. (2005)

A) Participants (n=60) were randomized using computer-generated pseudorandom numbers with variable block sizes. Procedure was adequately described and appropriate.

B) Allocation was concealed using serially numbered, opaque, sealed envelopes prepared by an administrative clerk not involved in the trial and kept in a locked cabinet.
C) The study was double-blind. Both anthocyanoside oligomer and placebo tablets were identical in appearance, with participants and study staff unaware of assignments.
D) Outcome assessments (mesopic contrast sensitivity with Visual Capacity Analyzer, structured symptom questionnaire) were performed by an investigator masked to treatment allocation.
E) All 60 randomized participants (30 per group) completed the 4-week study. No losses to follow-up or protocol deviations were reported.
F) Prespecified outcomes (contrast sensitivity, asthenopia questionnaire) were reported in full. There is no indication of missing or selectively reported outcomes.
H) The trial was conducted at Yonsei University with the investigational product Eyezone (Hanmi Pharmaceuticals, Seoul) provided by the manufacturer. Potential sponsor influence cannot be excluded. Additionally, slight imbalances in baseline refractive error and sex distribution were noted, which may introduce residual confounding.

<u>Lin et al. (2025)</u>
A) Participants (n=44) were randomized into intervention and placebo groups using a double-blind randomized design. The flowchart and methods describe random allocation, though specific software for sequence generation is not detailed beyond randomization.
B) Intervention and placebo tablets were identical in appearance, packaging, and taste, provided by TCI Co., Ltd., ensuring concealment of allocation from investigators and participants.
C) The study was explicitly double-blind. Both participants and research staff administering the intervention were unaware of group allocation. Identical chewable tablets maintained blinding.
D) Binocular accommodative facility (BAF) and amplitude of accommodation (AA) were measured using standardized clinical methods by masked assessors. No indication that outcome evaluators were unblinded.
E) Out of 52 recruited, 44 participants were analyzed. Dropout rate was ~15% (low compliance or lost to follow-up), balanced across groups and with reasons provided. No evidence of differential attrition affecting outcomes.
F) The trial was registered at ClinicalTrials.gov (NCT04348110). Prespecified outcomes (BAF, AA) were reported in full. No evidence of unreported outcomes.
H) The study product was designed, produced, and supplied by TCI Co., Ltd., raising potential conflicts of interest related to sponsorship and industry involvement. Although declared, this may introduce bias. Sample size was relatively small (n=44), which could limit robustness of findings.

<u>Mori et al. (2019)</u>
A) Randomization performed using block randomization stratified by sex, age, and refraction. Process is clearly described and appropriate.
B) Capsules were identical in appearance and packaging, and allocation was handled by an independent third party who sealed and assigned experimental drugs.
C) Explicitly double-blind trial. Both placebo and crocetin capsules were indistinguishable, ensuring participants and clinical staff were masked.
D) Examiners (orthoptists and ophthalmologists) were masked to group allocation. Outcomes (axial length, cycloplegic SER, OCT choroidal thickness) were objective, reducing detection bias.
E) 69 children randomized, 67 completed (2 dropouts: 1 noncompliance, 1 scheduling issue). Dropouts were few (<5%) and balanced. Reasons documented, no evidence of bias.

F) Trial registered (JapicCTI-173777), prespecified outcomes (axial length, refraction) were fully reported. Additional exploratory outcome (choroidal thickness) also transparently reported. No evidence of selective reporting.
H) The study was funded by ROHTO Pharmaceutical Co., Ltd., which also holds a patent (No. 6502603) on crocetin therapeutic applications. Several authors acknowledge support from ROHTO staff. This introduces a clear risk of sponsor-related bias. In addition, no sample size calculation was performed (pilot study design), which may increase risk of small-study bias.

Solé et al. (1984)
A) The study is described as a controlled clinical trial, but the method used to generate the sequence (random, quasi-random, or other) is not specified. Without explicit details, randomization cannot be confirmed.
B) No description of whether treatment allocation was concealed from investigators or patients during assignment. Potential risk that allocation was predictable.
C) The trial does not specify whether blinding was implemented. Since outcomes included visual acuity tests, lack of blinding could influence performance or reporting, although objective measures (electroretinograms) are less prone to this bias.
D) The study reports electro-oculogram and electroretinogram measurements, which are largely objective, but no explicit statement about masking of examiners is provided.
E) A total of 31 patients were enrolled, and results are reported for the full sample. No losses to follow-up or exclusions are mentioned, suggesting outcome data were complete.
F) Outcomes relevant to visual acuity and electrophysiological testing were reported. However, there is no trial registration or protocol, so selective reporting of results cannot be ruled out.
H) Very small sample size (n=31) with unclear methods of randomization and blinding. Also, limited reporting standards (pre-CONSORT era) increased risk of methodological bias. Sponsorship or manufacturer involvement is not declared but cannot be excluded.

Tanito et al. (2012)
A) Participants were assigned to lutein or zeaxanthin groups using a computer-generated random number table, clearly described in the methods.
B) Capsules were identical in appearance and prepared by an external provider. Investigators and participants had no access to group codes until the end of the study.
C) The study was double-blind. Both participants and study staff were unaware of group assignments, and capsules were indistinguishable.
D) MPOD measurements were conducted by trained technicians masked to treatment allocation, minimizing bias in outcome assessment.
E) All participants completed the 3-month follow-up. Some AFI measurements were missing due to device maintenance, not related to group allocation, with balanced missingness across groups.
F) All prespecified outcomes (MPOD assessed by RRS and AFI at defined time points) were reported in results. No evidence of outcome omission.
H) Ethical approval obtained, baseline characteristics comparable, no major sources of bias identified. A potential issue is that one author holds patents for the devices (RRS and AFI), but this is transparently declared and unlikely to affect supplement efficacy results.

Yoshida et al. (2023)
A) Randomization was performed using stratified random numbers generated by statisticians with SAS software. This method is clearly reported and appropriate.

B) Allocation was managed with drug numbering and blinding staff. Study capsules (lutein and placebo) were manufactured and provided by Santen Pharmaceutical, identical in appearance, and coded until data lock.
C) This was a double-blind RCT. Participants, investigators, and study staff were blinded to group assignment. Capsules were indistinguishable.
D) MPOD, BCVA, contrast sensitivity, and ERG were measured by technicians/investigators who were masked to treatment allocation.
E) Of 44 randomized patients (22 per group), only 28 (64%) completed per protocol (15 lutein, 13 placebo). Some participants refused continuation or had poor compliance. Reasons for dropout were documented, but attrition was relatively high, and analyses were limited to the per-protocol set. No intention-to-treat analysis was reported.
F) The trial was registered (jRCT ID: jRCTs031180168). Prespecified outcomes (MPOD, VA, CS, ERG, safety) were reported at all time points. No evidence of selective reporting.
H) The trial was funded by Santen Pharmaceutical Co. Ltd., and one co-author was an employee of the sponsor. The authors state the sponsor did not influence analysis or reporting, but industry involvement may introduce potential bias. Additionally, the sample size was small, and follow-up was short (6 months).

Zhang et al. (2022)
A) Subjects were randomly assigned to five groups (low-/high-dose Lycium barbarum, low-/high-dose lutein, blank control) using a validated random number generator, as reported in the methods.
B) While randomization was described, there is no clear description of allocation concealment (e.g., sealed envelopes or independent drug coding). Thus, the risk of selection bias cannot be excluded.
C) The authors explicitly state that blinding was not implemented because of anticipated compliance issues. Participants and study personnel knew the assigned interventions, which could influence outcomes (particularly symptom-related assessments and adherence).
D) MPOD was measured using heterochromatic flicker photometry (MPSII®), a psychophysical test dependent on participant response. Without assessor blinding, there is a substantial risk that knowledge of intervention could bias outcomes.
E) A total of 96 eyes from 54 participants were enrolled, and all completed the 3-month follow-up. No missing outcome data were reported.
F) The trial was registered in the Chinese Clinical Trial Registry (ChiCTR2100046748). Prespecified outcomes (MPOD at baseline and 3 months, subgroup analyses) were fully reported.
H) The trial was funded by the Beijing Traditional Chinese Medicine Technology Development Fund Project (JJ2018-50). No conflicts of interest were declared, but potential bias may arise from short follow-up (3 months), relatively small sample size (n=54), and reliance on subjective MPOD measurement techniques.
