# Supplementary figures and images for: Nutritional Supplementation for Myopia Prevention and Control: A Systematic Review of Randomized Controlled Trials
Source: Nutrients. 2025 Dec 19;18(1):4. doi: 10.3390/nu18010004 (PMC12787848; doi:10.3390/nu18010004)

**Additional file S4:** Funnel Plot of Included RCTs

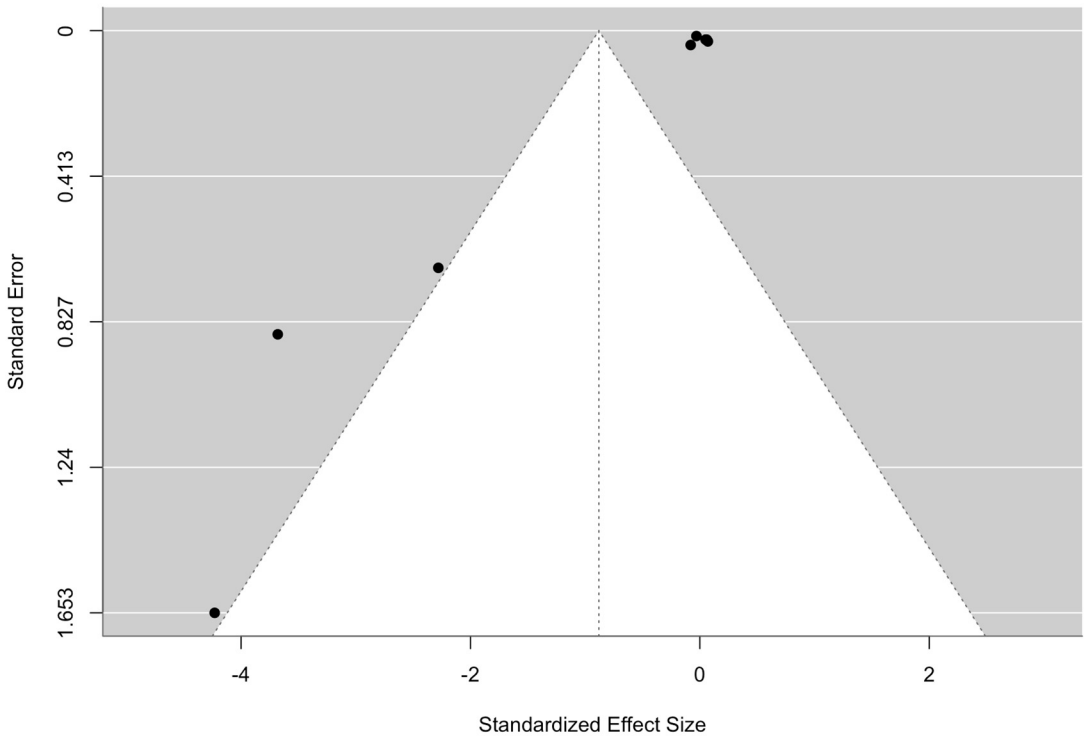

Supplement: Supplementary file 1 [file nutrients-18-00004-s001.zip › nutrients-4049080-supplementary/Additional file S4.pdf]
